# The intestinal distribution pattern of appetite- and glucose regulatory peptides in mice, rats and pigs

**DOI:** 10.1186/s13104-016-1872-2

**Published:** 2016-02-02

**Authors:** Nicolai J. Wewer Albrechtsen, Rune E. Kuhre, Signe Toräng, Jens J. Holst

**Affiliations:** NNF Center for Basic Metabolic Research and Department of Biomedical Sciences, Panum Institute, Faculty of Health and Medical Sciences, University of Copenhagen, Blegdamsvej 3B, 12.2, 2200 Copenhagen N, Denmark

**Keywords:** Gut hormones, Gastrointestinal tract, Distribution of gut hormones in the mouse rat and pig

## Abstract

**Background:**

Mice, rats, and pigs are the three most used animal models when studying gastrointestinal peptide hormones; however their distribution from the duodenum to the distal colon has not been characterized systematically across mice, rats and pigs. We therefore performed a comparative distribution analysis of the tissue content of the major appetite- and glucose regulatory peptides: glucose-dependent insulinotropic polypeptide (GIP), glucagon-like peptide-1 (GLP-1), glucagon-like peptide-1 (GLP-2), oxyntomodulin/glicentin, neurotensin, and peptide YY (PYY) from the duodenum to distal colon in mice (n = 9), rats (n = 9) and pigs (n = 8), using validated radioimmunoassays.

**Results:**

GLP-1, GLP-2 and oxyntomodulin/glicentin show similar patterns of distribution within the respective species, but for rats and pigs the highest levels were found in the distal small intestine, whereas for the mouse the highest level was found in the distal colon. In rats and pigs, neurotensin was predominantly detected in mid and lower part of the small intestine, while the mouse showed the highest levels in the distal small intestine. In contrast, the distribution of GIP was restricted to the proximal small intestine in all three species. Most surprisingly, in the pig PYY was found in large amounts in the proximal part of the small intestine whereas both rats and mice had undetectable levels until the distal small intestine.

**Conclusions:**

In summary, the distribution patterns of extractable GIP, GLP-1, GLP-2, oxyntomodulin/glicentin, neurotensin are preserved across species whereas PYY distribution showed marked differences.

**Electronic supplementary material:**

The online version of this article (doi:10.1186/s13104-016-1872-2) contains supplementary material, which is available to authorized users.

## Background

The gastrointestinal tract plays a key role for the regulation of appetite and blood glucose [1–3]. This is partly due to the secretion of the proglucagon (pcg) derived gut hormones: glucagon-like peptide 1 (GLP-1), glucagon-like peptide 2 (GLP-2), oxyntomodulin/glicentin; as well as non-pcg derived peptides: glucose-dependent insulinotropic peptide (GIP), neurotensin (NT), and peptide YY (PYY), all of which inhibit appetite [2], while some, in addition, potentiate glucose-stimulated insulin secretion (GIP and GLP-1) or suppress glucagon secretion (GLP-1) [4]. The distribution of gut hormones along the gastrointestinal tract in mice, rats, and pigs has primarily been assessed by immunohistochemistry and/or messenger RNA (mRNA) determination, but these may not always be reliable surrogate markers for protein content and thereby secretion [5]. Potential discrepancies may therefore be related to the validity of the method used for assessing the distribution of appetite- and glucose regulating peptides. In the case of immunohistochemistry, the specificity of antisera used [6], the tissue preparation, antigen retrieval, thresholds for positive and negative signal may interfere with interpretation of intestinal distribution patterns [7–9]. With regards to the validity of mRNA data this indeed depends on primer specificity but also on post-transcriptional regulation such as the regulation by micro RNAs [10, 11], and of course also on the usual reliability criteria regarding precision/sensitivity and accuracy.

Mice, rats, and pigs are the three most used model animals in enteroendocrine research [12], but their resemblance to one another with respect to their intestinal distribution of appetite- and glucose regulatory peptides has not been investigated systematically [13]. In the current study we used well characterized radioimmunoassays, previously validated according to the guidelines by Clinical and Laboratory Standards Institute [14–19], to perform an in-depth distribution analysis of six appetite- and glucose regulatory peptides, hypothesizing that the distribution profiles for these peptides may differ across species due to their different patterns of feeding; rodents being “nibblers” and coprophagic, while pigs like humans are omnivores and tend to eat fewer and larger meals.

## Methods

### Tissue

Studies were carried out with permission from the Danish Animal Experiments Inspectorate (2013-15-2934-00833) and in accordance with the EU Directive 2010/63/EU and guidelines of Danish legislation governing animal experimentation (1987) and the National Institutes of Health (publication number 85-23). Tissue was harvested at 8–11 AM for all species which were obtained during previous study [20] investigating the amidation efficiency of GLP-1. Non-fasted Wistar rats (weight = 274.3 ± 11.7 g; n = 9) (Taconic, Ejby, Denmark) were anesthetized by subcutaneous injection with Hypnorm^®^/Midazolam^®^ (0.2 ml/100 g, conc. 5 mg/ml) and tissue samples (≈1.5 cm/piece) were collected from duodenum, proximal jejunum, distal ileum, caecum, proximal colon and distal colon. Thereafter, the rats were euthanized by diaphragm perforation. Non-fasted male C57bl/6 mice (weight = 30 ± 5 g; n = 9) (Charles River Laboratories International, Inc., Erkrath, USA) were sacrificed by cervical dislocation and tissue samples (≈3 cm/piece) were collected from regions corresponding to those of the rats. Porcine tissue was collected from animals of the LYY strain n = 8). Food was withdrawn 24 h before surgery, but animals had free access to drinking water. After premedication with ketamine and induction with pentobarbital, animals were anesthetized with α-chloralose (66 mg/kg) and ventilated with intermittent positive pressure. Tissue (1 cm/piece) was collected from same regions as the rat and mouse. The pigs were sacrificed by pentobarbital. For all species, all tissues were thoroughly rinsed in cold PBS, snap frozen on dry ice and stored at −80 °C until protein extraction.. The anatomical definitions in mice, rats and pigs are shown in Table 1.

**Table 1:** Definitions of intestinal segments

	Wistar rat	C57bl/6/mouse	LYY strain pig
Duodenum	0.5 cm after pyloric sphincter	0–3 cm after pyloric sphincter	5 cm after pyloric sphincter
Prox. Jejunum	6 cm after pyloric sphincter	3 cm after pyloric sphincter	50 cm after pyloric sphincter
Dist. ileum	0.5 cm before the ileo-cecal junction	0.5 cm before the ileo-cecal junction	10 cm before the ileo-cecal junction
Caecum	Mid part of caecum	Mid part of caecum	Mid part of caecum
Prox. colon	0.5 cm after colon connects to caecum	First 5 cm of colon	10 cm after caecum
Dist. colon	0.5 cm before rectum	0.5 cm before rectum	10 cm before rectum

The anatomical definitions shown above have previously been published by Kuhre et al. [20]

In present study we tested two different protein extraction methods (trifluoroacetic acid and formic acid), several reconstitution buffers and dilution factors for optimization of the subsequent measurements by radioimmunoassays. The extracts were purified using pH resistant tc18 cartridges (Cat. no. WAT036810, Waters, MA, USA), dried under a gentle stream of compressed air overnight and reconstituted in 1 mL of TRIS buffer; 100 mmol/l TRIS buffer (Cat. no. T-3253 and T1503, Sigma Aldrich) supplemented with 0.1 % (w/v) human serum albumin (Cat. no. 12666, Merck KGaA, Darmstadt, Germany), 20 mmol/l EDTA and 0.6 mmol/l Thimerosal (Cat. no. T-5125, Sigma Chemical Co., St. Louis, MO, USA) (pH = 8.5), and further diluted in assay buffer to ensure that the measured concentrations were within the detection range of the respective standard curves.

### Biochemical measurements

Amidated GLP-1, intact GLP-2, total neurotensin, total PYY, and intact GIP levels were measured with well characterised in-house developed RIAs validated according to the Clinical and Laboratory Standards Institute Protocols as described [17, 19–22]. For simultaneous measurements of oxyntomodulin/glicentin we used an in-house developed C-terminus specific RIA assay with equal recoveries (98 ± 11 %) of oxyntomodulin and glicentin as described previously [14]. The RIAs have inter-assay variations of <15 %, intra-assay variations of 5–10 % and their specificity, sensitivity and precision has been tested according to the criteria of Richterich as described [15, 16].

### Statistics analysis

Data are presented as mean relative fold variation ±1 SEM, relative to the overall average of peptide content across (pmol/g) all intestinal segments in the respective species. Raw data (pmol/g) are shown in Additional file 1: Table S1. Overall average was calculated on the basis of the measured peptide concentrations, the dilution factor employed and the weight of the respective biopsies and expressed in pmol/g. Differences in segmental hormone concentrations were evaluated by one-way ANOVA for repeated measurements corrected for multiple testing by Bonferroni post hoc analysis. P < 0.05 was considered significant. Prior to the statistical test, normality and homoscedasticity in datasets were tested by both Shapiro–Wilk test (swilk command) and visually from residual plots. Calculations and figures were prepared using GraphPad Prism, GraphPad Software, La Jolla California USA.

## Results

### GLP-1, GLP-2 and oxyntomodulin/glicentin

**Fig. 1:**
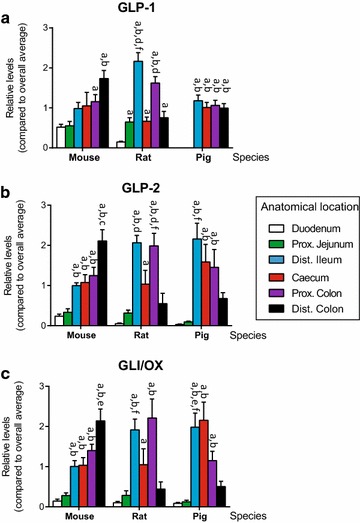
Tissue concentration of GLP-1, GLP-2 and GLI/OX in mouse, rat and pig. Distribution of proglucagon derived peptides along the gastrointestinal tract in mice, rats and pigs. Data are shown as relative means ± 1SEM (compared to overall average for the respective species). **a** GLP-1 total, **b** GLP-2 (intact) and GLI/OX (total) for duodenum (*D*), proximal jejunum, (*PJ*), distal ileum (*DI*), caecum (*C*), proximal colon (*PC*) and distal colon (*DC*). Significance is indicated above respective *bars*. Significant different (P < 0.05) from **a** duodenum, **b** proximal jejunum, **c** distal ileum, **d** caecum, **e** proximal colon and **f** distal colon

Levels of amidated GLP-1 increased significantly (Fig. 1a, P < 0.01) along the gastrointestinal tract in the mouse, whereas in the rat the highest levels were found in the distal ileum. In the pig, similar concentrations were found from distal ileum to distal colon, but nothing was detected in the duodenum or the proximal jejunum (Fig. 1a). GLP-2 showed similar patterns of distribution as GLP-1, although for the pig, GLP-2 levels were significantly lower (Fig. 1b, P < 0.05) in the colon compared to the small intestine. Oxyntomodulin/glicentin patterns paralleled GLP-2 levels in all three species (Fig. 1c).

### GIP, neurotensin and PYY

**Fig. 2:**
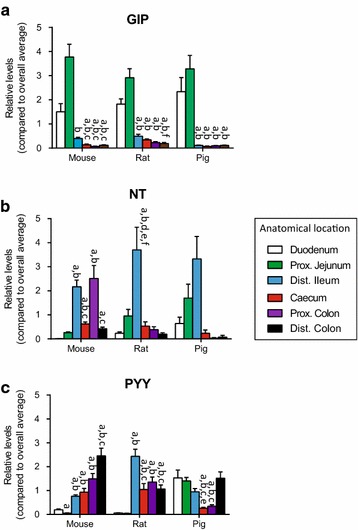
Tissue concentration of GIP, NT and PYY in mouse, rat and pig. Data are shown as relative means ± 1SEM (compared to overall average for the respective species). **a** GIP total, B: NT (intact) and PYY (total) from duodenum (*D*), proximal jejunum, (*PJ*), distal ileum (*DI*), caecum (*C*), proximal colon (*PC*) and distal colon (*DC*). Significance are indicated above respective *bars*. Significant different (P < 0.05) from **a** duodenum, **b** proximal jejunum, **c** distal ileum, **d** caecum, **e** proximal colon and **f** distal colon

Profiles of GIP levels along the gastrointestinal tract were similar across all three species (Fig. 2a). Highest concentrations were found in the proximal jejunum with decreasing concentrations in the subsequent anatomical segments down the intestine. In contrast, the concentrations of neurotensin varied between mouse, rat and pig (Fig. 2b). In the rat and pig, highest levels were found in the distal ileum, whereas the mouse had similar levels in the distal ileum and the proximal colon. PYY levels continued to increase along the gastrointestinal tract of the mouse, with highest levels in the distal colon, whereas in the pig, PYY levels are shifted towards the small intestine (Fig. 2c). Finally, in the rat, PYY levels were below detection in the proximal small intestine (duodenum and proximal jejunum) with the highest levels found in the distal ileum and about half of that in the remaining anatomical sections.

## Discussion

Our analysis demonstrates that while distribution of GLP-1, GLP-2, oxyntomodulin/glicentin, neurotensin, and GIP is relatively preserved across species, the anorectic hormone PYY, to our surprise, showed marked interspecies differences (Figs. 1, 2). In contrast, previous studies using gene expression, immunohistochemistry or RIA reported low (or no) expression of PYY in the proximal small intestine in the pig [13, 23, 24]. However, Cho et al. recently suggested that differences across pig strains may occur [25]. We are unable to determine from the present results whether this is relevant for our data (with material obtained from pigs of the Danish LYY/LYD strain). For the rat, the peptide was undetectable in the proximal small intestine (Table 1), in both diluted and undiluted extracts (data not shown for latter), and concentrations increased from distal ileum to distal colon. Based on the sensitivity of the assay used for measuring PYY (lower limit of detection ~1.5 ± 0.1 pmol/l) and a post hoc power calculation (one sided student t test, comparing the reference values and variations to the lower limit of detection with alpha value of 0.05 yielding an estimated a power of 89.75 %), we find it unlikely that this observation is the result of a type 2 error. The rat also had no detectable PYY stored in the proximal small intestine whereas the highest concentration was found in distal ileum, although appreciable concentrations were also detected in the large intestine. This is partly in agreement with other studies that have found increasing amounts of PYY distally along the gastro-intestinal tract in man, monkey, rat and mouse [5, 24–29]. Interestingly, the pig showed a complete different pattern of PYY storage compared to the mouse and rat (Fig. 2) and to that described for humans [29]. In pigs, PYY was stored in the entire small intestine in concentrations that were comparable to that found in distal colon, while very little was detected in the caecum and proximal colon.

With regards to the distribution pattern of the proglucagon derived peptides: GLP-1, GLP-2 and oxyntomodulin/glicentin, all three of them closely mirrored each other (equimolar amounts), as would be expected given that they are derived from the same precursor (although prohormone processing might differ) [30]. Nevertheless, in the pig, GLP-2 levels decreased along the colon compared to the mouse and rat. To our knowledge, this has not been reported in other studies [31]. Similar findings, although not as extensively examined, have been reported for humans [32]. However, while the concentration of these peptides progressively increased from duodenum towards distal colon in the mouse, the highest concentrations in rat and pig were found in distal ileum, caecum, and proximal colon with little or no detectable concentrations in proximal small intestine. A similar, scattered distribution of GLP-1, GLP-2, and oxyntomodulin/glicentin has previously been reported using immunohistochemistry [23, 33].

In rats and pigs, neurotensin was predominantly stored in the mid and distal part of the small intestine with little or no measureable peptide in the large intestine (Fig. 2), while the mouse had the highest neurotensin concentrations in the distal ileum and proximal colon, in agreement with another study in rats and humans [34]. Our study does have several limitations: we did not characterize the cellular origin of the peptides of interest and we cannot exclude that some variation may exist between strains of the tested animals. Therefore we chose to characterize frequently used strains of the respective animals to increase the relevance of our findings.

In summary, the mouse conforms with the classical assumption that levels of enteroendocrine hormones increase along the gastrointestinal tract [35], whereas the rat and particular the pig may differ—at least when estimated by quantification of extractable tissue peptide contents. However, also peptide content may not necessary reflect secretory output/capacity, as indicated by a recent study from our group [5]. Another important observation in this study is that while the tissue concentrations of GLP-1 and PYY parallel each other closely in the mouse and rat, this was not the case for the pig. In this case, high concentrations of PYY are found in the proximal small intestine where little or no GLP-1 was detected. It is generally believed that PYY is co-produced and co-secreted with GLP-1 in distal L-cells of the small intestine and large intestine in rats [5, 26], mouse [27, 36] and humans [27, 32, 37]. Given that GLP-1 excursions typically peak around 30–60 min after meal intake in humans (and faster in rodents), it remains a mystery why the concentration of GLP-1 is so high in the distal part of the gastrointestinal tract. Several hypotheses exists; both GLP-1 and GLP-2 have been shown to enhance growth of the intestinal mucosa [38], suggesting that the high distal concentration of these peptides may play a role in the adaptative regulation of the growth of the colonic epithelium. Based on the observation that GIP can stimulate GLP-1 secretion from the L-cell model line GLUTag it has been suggested that the distally located L-cell may be activated by circulating GIP (which are rapidly released upon meal intake), thereby enabling distally located L-cells to contribute to the GLP-1 response long before luminal chyme has reached these cells [39]. However, in perfused pig ileum, only supra-physiological concentrations of GIP stimulated GLP-1 secretion [40] and even rather high doses of GIP do not stimulate GLP.1 secretion in humans [41]. Noteworthy, our study, however, indirectly indicates that, at least in case of the LYY/LYD pig, PYY may be produced by a different enteroendocrine cell than the classical L-cell. Finally, it would be of substantial interest to determine which of the three species has the “highest” similarity to humans in respect distribution of gut hormones along the GI tract. Unfortunately, to the best of our knowledge, currently, a similar comparative human study on the distribution of gut hormones along the gastro-intestinal tract does not exist. Therefore, the only insight that can be gained on this matter depends on scanty evidence from 30 years old literature [35, 42], where analytical specificity may have been questionable. Future studies investigating the human gastrointestinal tract by both mRNA, peptide content, and immunohistochemistry may provide new and important knowledge of the complex endocrine system allowing a qualified decision regarding the best animal model for humans. Taken together, our findings conform to our hypothesis; that the distribution of glucose and appetite regulating hormones are similar in rodents (rats and mice) but different in pigs, in particular in respect to the proximal small intestine. Perhaps these differences may be explained by variance in feeding patterns observed between rodents and pigs.

## Additional file

10.1186/s13104-016-1872-2 Measured concentrations.

## Abbreviations

GIP: glucose-dependent insulinotropic peptide
GLP-1: glucagon-like peptide 1
GLP-2: glucagon-like peptide 2
PYY: peptide YY
NT: neurotensin
RIA: radioimmunoassay
